# Supplementary material for: Very high density EEG elucidates spatiotemporal aspects of early visual processing
Source: Sci Rep. 2017 Nov 24;7:16248. doi: 10.1038/s41598-017-16377-3 (PMC5701165; doi:10.1038/s41598-017-16377-3)
Supplement: Supplementary file 1 — Supplementary Material [file 41598_2017_16377_MOESM1_ESM.doc]

**Very high density EEG elucidates spatiotemporal aspects of early visual processing**

Amanda K Robinson^1,2,*^, Praveen Venkatesh^2,3^, Matthew J. Boring^2,4^, Michael J. Tarr^1,2^, Pulkit Grover ^2,3,#^, Marlene Behrmann^1,2,#^

^1^ Department of Psychology, Carnegie Mellon University

^2^ Center for the Neural Basis of Cognition, Carnegie Mellon University

^3^ Department of Electrical and Computer Engineering, Carnegie Mellon University

^4^ Department of Neuroscience, University of Pittsburgh

^#^ Shared contribution

* Correspondence to Amanda K. Robinson: [amanda.robinson@uqconnect.edu.au](mailto:amanda.robinson@uqconnect.edu.au)

**Supplementary material**

S1. Decoding performance per stimulus for time course analysis

SND electrodes provided substantial improvement in overall decoding accuracy over equivalent ND arrays. To assess whether this SND benefit was equivalent for classification of all six stimuli, we analyzed performance of the classifier in terms of the confusion matrix. As an index of performance per stimulus, the F measure (the harmonic mean of the recall and precision of the classifier) was calculated. As can be seen in Figure S1, the time course of performance for each stimulus was similar to that of the overall classification accuracy, with peaks at approximately 100 ms. At the mean of each participant’s peak, a 5 x 2 x 3 repeated measures ANOVA with factors of electrode array, stimulus visual field (left, right) and stimulus spatial frequency (low, medium, high) revealed a significant main effect of electrode array, *F*_4,60_ = 9.98, *p* < .001, *η_G_^2^* = .028, such that SND outperformed all ND configurations, *t*s > 3.33, *p*s < .005, *g*_av_ > .336. There was also a significant effect of stimulus spatial frequency, such that high SF stimuli had higher classification accuracy than medium and low SF stimuli, *p*s < .001. Interestingly, there was a significant interaction between spatial frequency and electrode array,  *F*_8,120_ = 2.16, *p* = .035, *η_G_^2^* = .006. To follow up on this interaction, paired *t*-tests were conducted across electrode arrays for each level of spatial frequency stimuli, using Bonferroni-Holm correction. For the high spatial frequency stimuli, SND had higher performance than all ND arrays, *p*s < .066. ND1 was also significantly lower than ND3, *p* = .016. For the medium spatial frequency stimuli, SND was marginally higher than ND1 and ND2, *p*s = .055. For the low spatial frequency stimuli, SND resulted in higher accuracy than ND1, *p* = .001, but there were no differences across the other arrays, *p*s > .105.

In sum, SND EEG resulted in substantially better classification than ND, particularly for the high spatial frequency stimuli. Of note, performance was better for high spatial frequency in the RVF than LVF (SND: *p* = .061, ND: *p* = .151), which is consistent with the left hemisphere having a preference for high spatial frequencies (Woodhead, Wise, Sereno, & Leech, 2011). These results indicate that our high spatial frequency stimuli lead to a neural representation with higher spatial frequency, which is better captured by SND EEG.


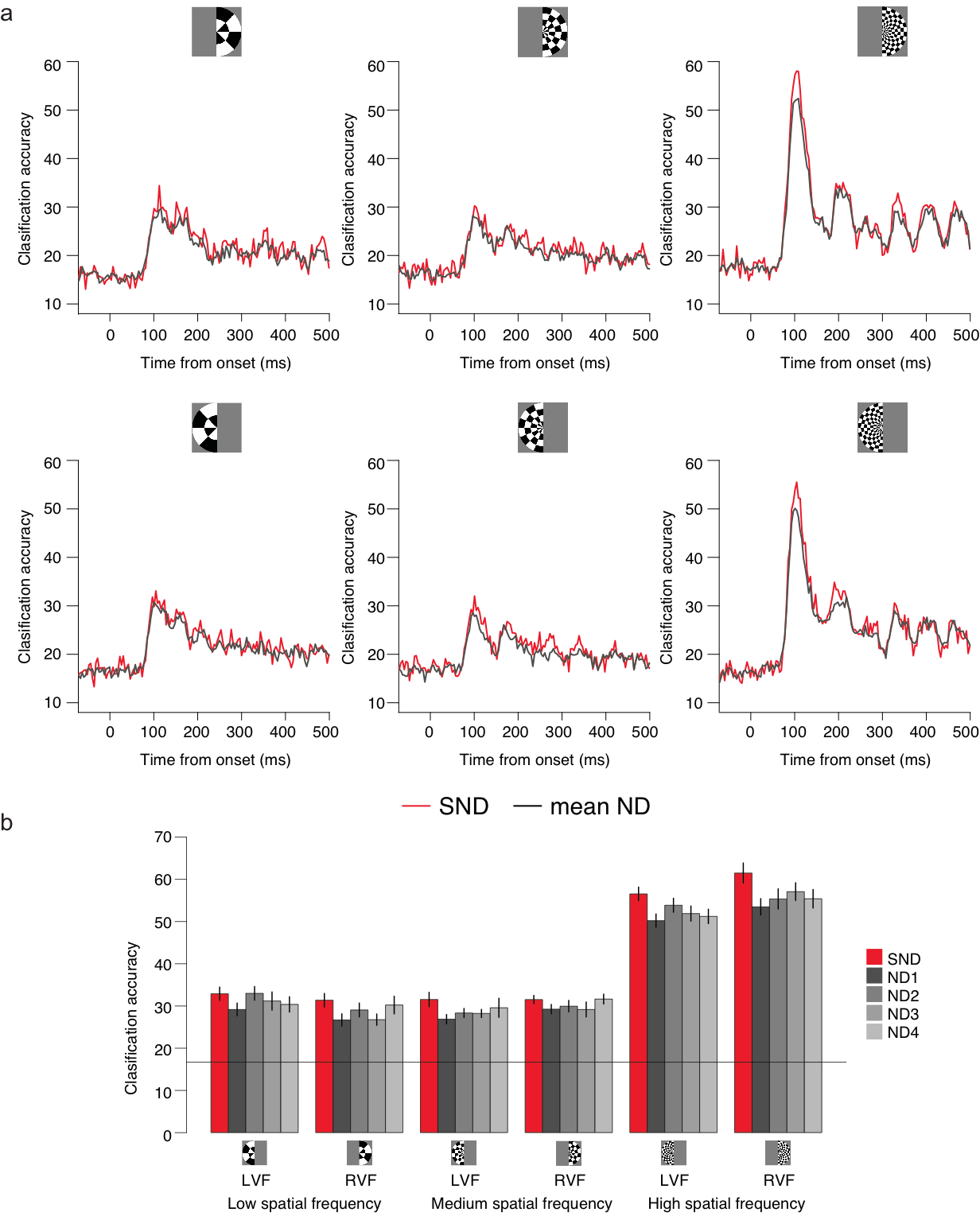


Figure S1. Classifier performance per stimulus. (a) Time course of classifier sensitivity per stimulus. Top row is RVF, bottom row is LVF stimuli. From left to right, columns indicate low, medium and high spatial frequency stimuli. (b) Average classifier performance per stimulus at peak decoding time per participant. SND outperformed ND arrays particularly for high spatial frequency stimuli.

S2. Time course decoding as a function of signal quality

One possible reason that SND outperformed ND is because of noise reduction during classification. If this were the case, electrodes with low SNR or higher noise might cause poor decoding performance for their associated ND configuration, but the SND array would not be impacted as much due to redundant information in electrodes surrounding the bad electrodes. To check if variance in signal quality influenced ND performance to a greater extent than SND, we assessed decoding accuracy for the ND configurations with the best and worst signal quality per participant. ND arrays with the fewest bad electrodes were compared with ND arrays with the greatest number of bad electrodes per participant. Electrodes were classified as bad if they would typically have be interpolated by the Prep pipeline(Bigdely-shamlo, Mullen, Kothe, Su, & Robbins, 2015), exceeded a voltage threshold of 150 μV on more than half the trials, or exceeded standard deviation of 5 for the grand average ERP of all trials (see RSA methods for details of excluded electrodes). For participants with equivalent numbers of “bad” or “good” electrodes in different arrays, the classification accuracy was taken as a mean between the equivalent arrays. On average, the SND array contained 7.56 bad electrodes (*SD* = 5.32), the best ND array contained 0.63 bad electrodes (*SD* = .72), and the worst ND array contained 3 bad electrodes (*SD* = 1.83). Decoding results for these modified electrode arrays can be seen in Figure S2. At the mean peak in decoding per participant, there was a significant main effect of electrode array, *F*_2,30_ = 14.02, *p* < .001, *η_G_^2^* = .047, such that SND significantly outperformed both the “best” and the “worst” ND arrays, *t*s > 4.27, *p*s < .001, *g*_av_ > .345. There was no significant difference between the best and worst ND arrays, *t* = 1.62, *p* = .125, *g*_av_ = .142. EEG signal quality is thus unlikely to be responsible for the SND superiority in this experiment.


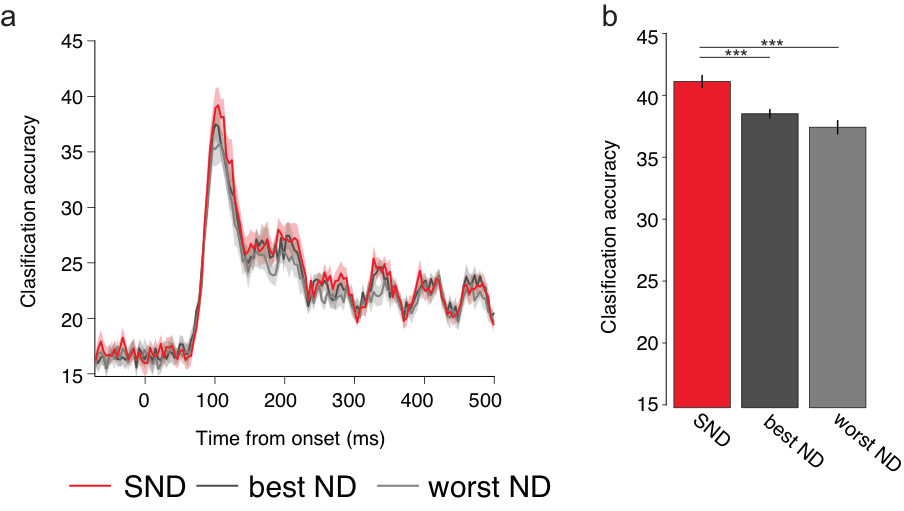


Figure S2. Decoding performance for SND and ND arrays, with the ND arrays split by signal quality per participant.  (b) Mean classification at 10 ms participant peak. SND outperformed both ND arrays, and there was no significant difference between the best ND and worst signal ND arrays.  **p* < .001.

S3. Dissimilarity data for ERP data

To construct the EEG dissimilarity measure, we calculated Euclidean distance for the voltage across electrodes for all pairwise comparisons of stimuli at each time point from image onset. These results revealed relatively stable distance values during the baseline period, which then sharply increased and peaked at 100 ms (Figure S3a). Dissimilarity diverged across the pairwise stimuli combinations during the peak. Euclidean distance also exhibited a periodic response, likely due to the periodic stimulus presentation. The trend was similar for the SND and ND electrode arrays. A 5 x 15 ANOVA on mean dissimilarity values at each participant’s individual peak revealed no significant effect of electrode array, *F*_4,60_ = 1.91, *p* = .120, *η_G_^2^* = .008. There was a significant main effect of stimulus pair, *F*_14,210_ = 36.30, *p* < .001, *η_G_^2^* = .667, but no significant interaction, *F*_56,840_ = .75, *p* = .910, *η_G_^2^* = .005 (Figure S3b).


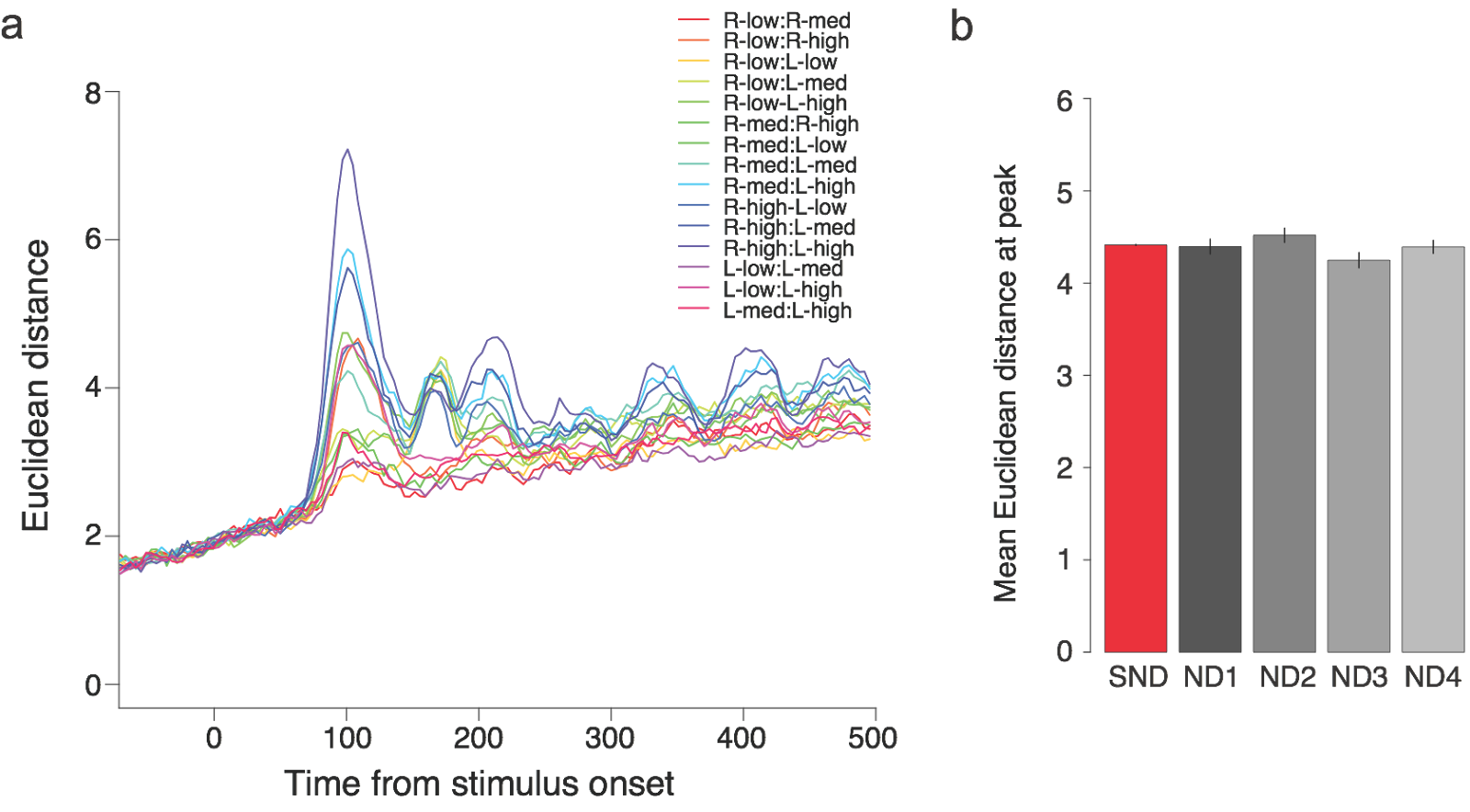


Figure S3. EEG stimulus dissimilarity. (a) Euclidean distance for each pair of stimuli across time for SND array. (b) Mean Euclidean distance per electrode set at first peak (mean across stimulus pairs and participants). There was no significant difference in dissimilarity values across electrode sets.

S4. RSA analyses with different layers of HMAX

In addition to the S1 layer of HMAX, RSA was conducted for layers C1, S2 and C2. These layers represent hierarchical processing in the visual system, such that C1 represents the local invariance maximum for features, S2 is an intermediate feature layer, and C2 is a global invariance layer, which represents the maximum response to the intermediate features across positions. RDMs computed from the outputs of each layer reveal that the S1, C1 and S2 had similar relative responses, with a dissociation between visual field and spatial frequency of the stimuli (Figure S4.1). C2, in comparison, as a location-invariant layer, only exhibited a difference for spatial frequency.

RSA for each HMAX model layer revealed similar time course for layers C1 and S2 to that of the S1 RSA, with significant correlations from approximately 75 ms (Figure S4.2). A FFT of the correlation time course further revealed signal at 15 Hz for these layers, indicating that the periodic image presentation was being processed to higher levels than just simple cell responses in V1 (as indexed by S1 correlations). The C2 model did not reach significance at any time point, although once again there was a 15 Hz signal, indicating that periodic images were being processed to the level of view-invariant responses in early visual cortex. Importantly, while the S1, C1 and S2 RDMs were highly correlated with each other (*r*s > .943. *p*s < .001), the C2 RDM did not correlate significantly with the S1, C1, or S2 RDMs (|*r*|s < .09, *p*s > .740), so the C2 RSA signal at 15 Hz is indicative of an independent neural process. This result - a small and non-significant correlation in the time domain that was clearly evident in the frequency domain - is striking in demonstrating that periodic visual presentation can result in greater power even using RSA analyses.

Analysis of the noise ceiling revealed that the HMAX S1 model explained the EEG RDM at the first peak as well as could be expected given the noise in the data. At each time point, the upper estimate of the noise ceiling was calculated by correlating each participant’s EEG RDM with the group mean RDM and taking the mean across participants. The lower estimate of the noise ceiling was calculated by correlating each participant’s EEG RDM with the mean of the group excluding that individual participant. Figure S4.3 shows the upper and lower noise ceiling plotted with correlation curves for the four HMAX layers, plotted separately for each EEG array. S1 reached the lower noise ceiling at the first peak, and then C1 and S2 reached the noise ceiling at approximately 150 ms. Interestingly, the C2 curve, although not significant, revealed a peak at approximately 125 ms, after the S1, C1 and S2 model peaks. This is consistent with the hierarchical nature of the four layers, with S1 representing the lowest level of visual processing, and C2 representing a more complex, later process. Critically, these data show that EEG can represent different levels of early visual processing. SND analyses revealed the largest correlations and greatest 15 Hz amplitude for the C1, S2 and C2 correlation curves, but these were not significantly different from the ND arrays, unlike the S1 layer analysis. This is likely because of the disproportionate advantage for SND EEG for high spatial frequency neural information, which is most likely to manifest for the earliest level of visual processing in V1, which is closest to the scalp, and therefore not subject to as much high spatial frequency decay.


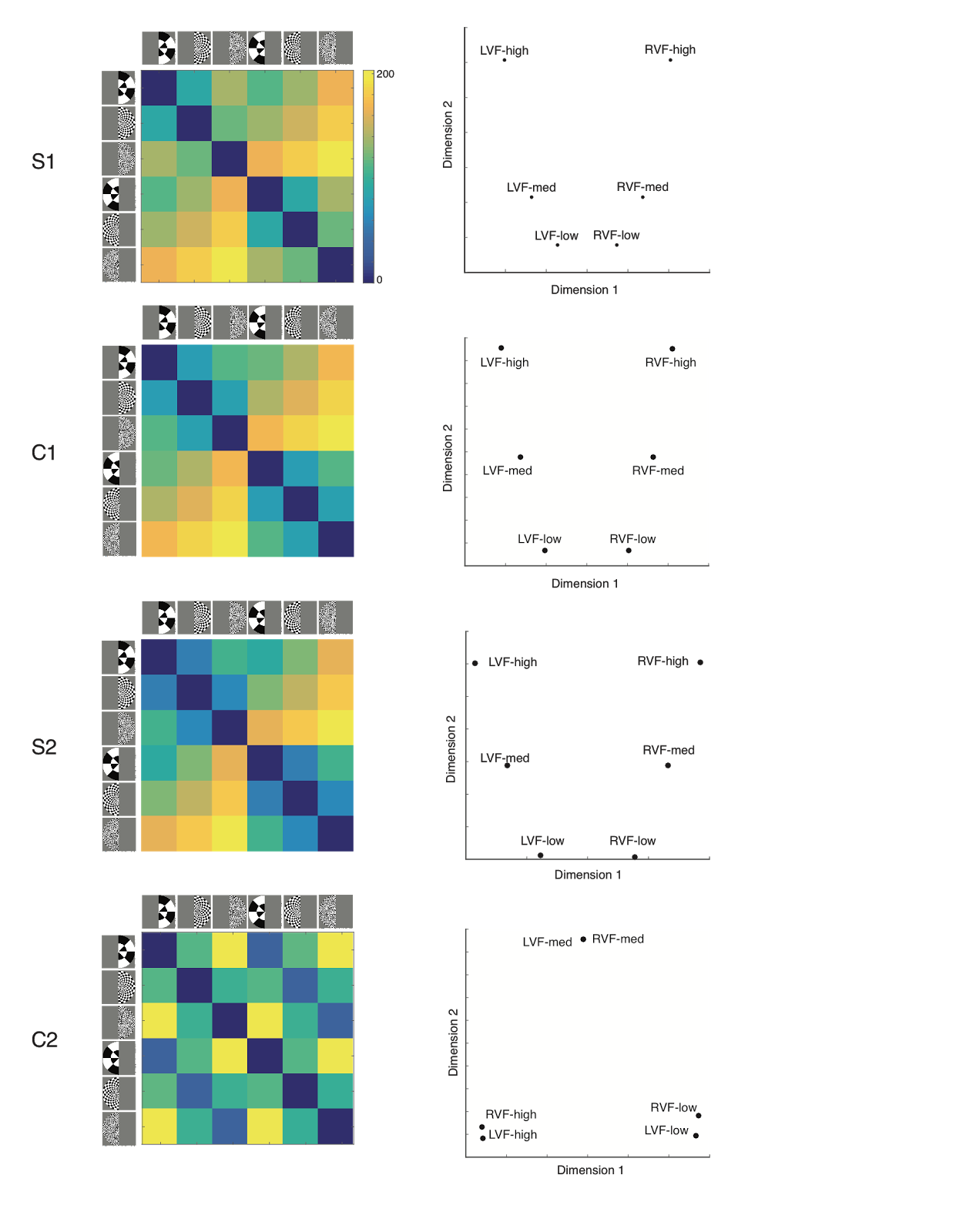


Figure S3.1 Representational dissimilarity matrices from (a) S1, (b) C1, (c) S2, and (d) C2 layers of the HMAX model for the experimental stimuli.


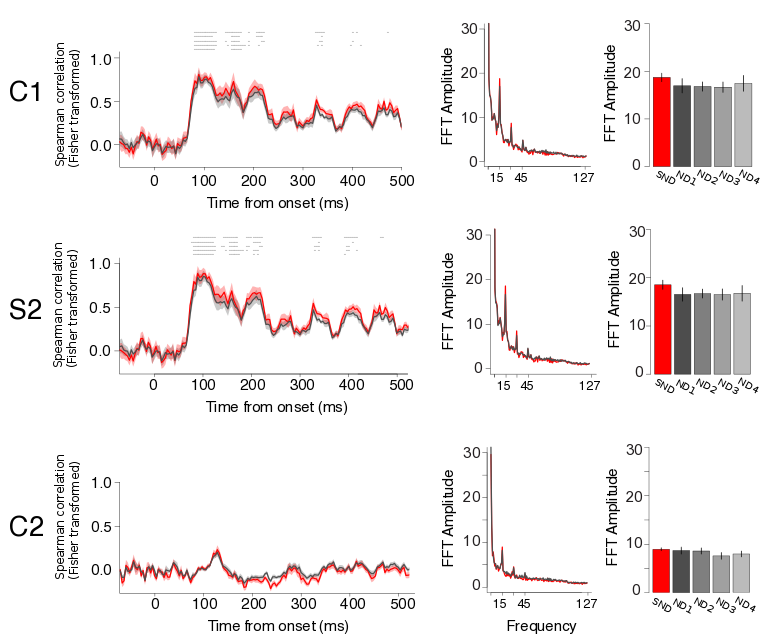


Figure S3.2. RSA analyses with HMAX layers C1, S2, and C2. Plots show the correlation time courses, the FFTs of the time courses, and the FFT amplitude at 15 Hz per layer. All layers revealed at 15 Hz signal, although C2 did not show significant groupwise correlation at any time point.


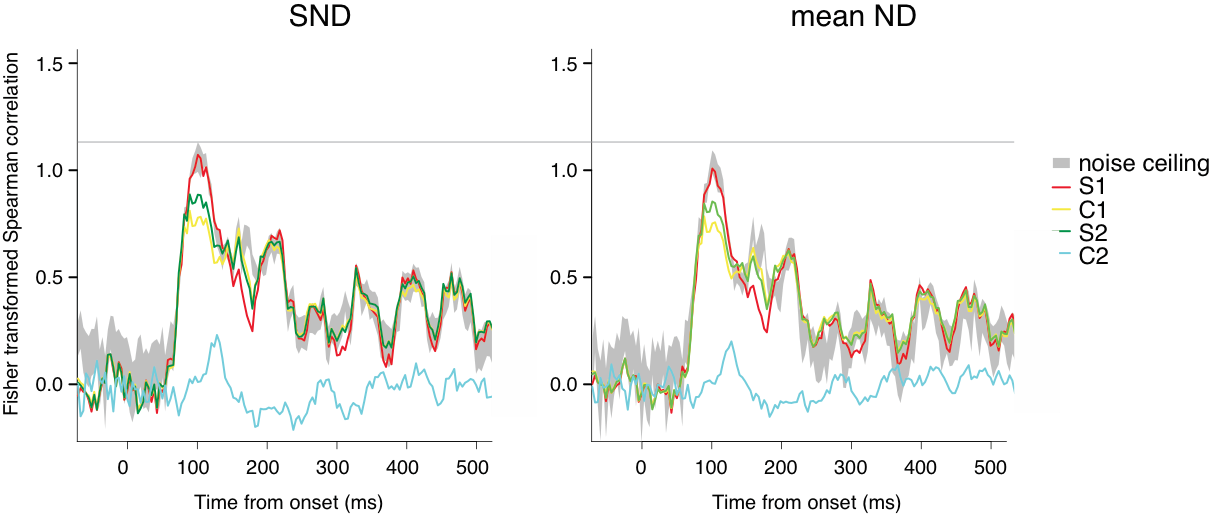


Figure S3.3. RSA analyses comparing RSA from first four HMAX layers with noise ceiling in the data for SND and mean ND arrays.

S5. Analyses with interpolated ND datasets

To assess if the different number of electrodes in the SND and ND arrays influenced the SND advantage for classification and RSA, the small ND arrays (30-33 electrodes) were interpolated to 128-electrode “ND” arrays. Interpolation was performed from the ND electrodes of the epoched data that was downsampled to 256 Hz. For each time point per trial, interpolation was performed using Biharmonic Spline Interpolation (Sandwell, 1987) (as implemented by the “griddata” function in Matlab) to extrapolate the ND arrays to 128 channels based on SND coordinates. Trial averaging and other processing steps were then performed as in the original analyses. Figure S5.1 depicts the mean voltage head maps at the critical time period, 100 ms post image onset, for the SND array and the four interpolated ND arrays per stimulus. It is evident that the SND array contains more high spatial frequency neural information.

We performed the time course classification, flicker classification and representational similarity analyses with the interpolated electrode arrays and compared them to the SND array. It is important to note that although the SND arrays in the current analyses had the exact same neural information as the original analyses, the decoding results were slightly different because different combinations of trials were used relative to the original analyses (pseudotrials fed to the classifier were formed by taking the mean of two random trials). The RSA analysis was exactly the same for the SND array.


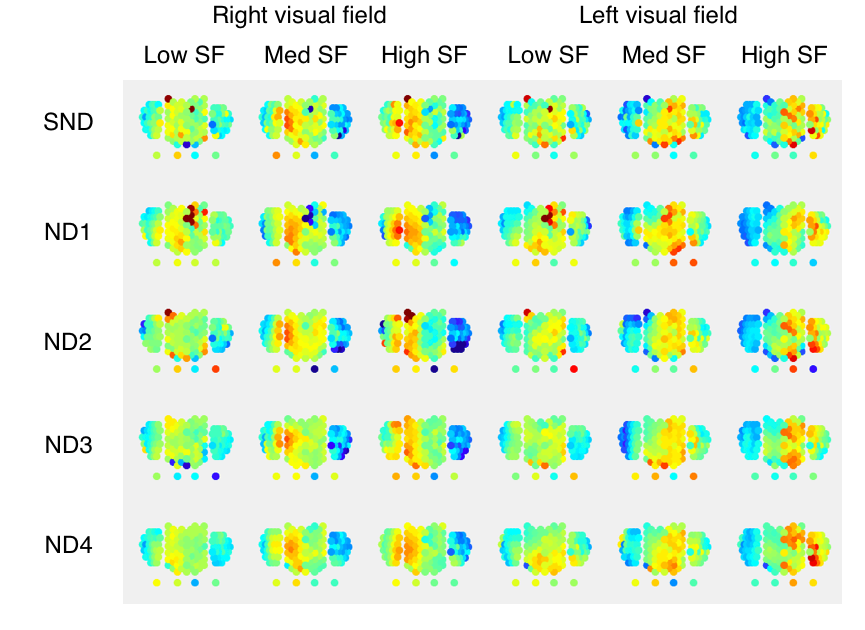


Figure S5.1 Voltage topography maps (2-dimensional) for each SND and ND array (rows) and stimulus (columns) at the time point 100 ms after image onset. The SND array shows the ERP values from the original 128-electrode grid, averaged across the 16 participants. The ND arrays depict the low-density electrode values interpolated to the 128-electrode grid. The head maps show contralateral positivity with respect to the stimulus. The SND array appears to represent neural information with higher spatial frequency.

*Time course classification*

Time course classification with the interpolated ND arrays was significantly poorer than with the SND array (see Figure S5.2). The interpolated ND results looked very similar to the results from the original, smaller ND sets. Principal components analysis was conducted on the electrodes at each time point, which detected the interpolated channels to be linearly dependent on the original electrodes and essentially weighted their contribution to zero. Thus, it is not surprising that, as in the original analysis, the SND classification was significantly higher than all ND arrays, *p*s < .005, and there were no significant differences between the ND sets, *p*s > .233.


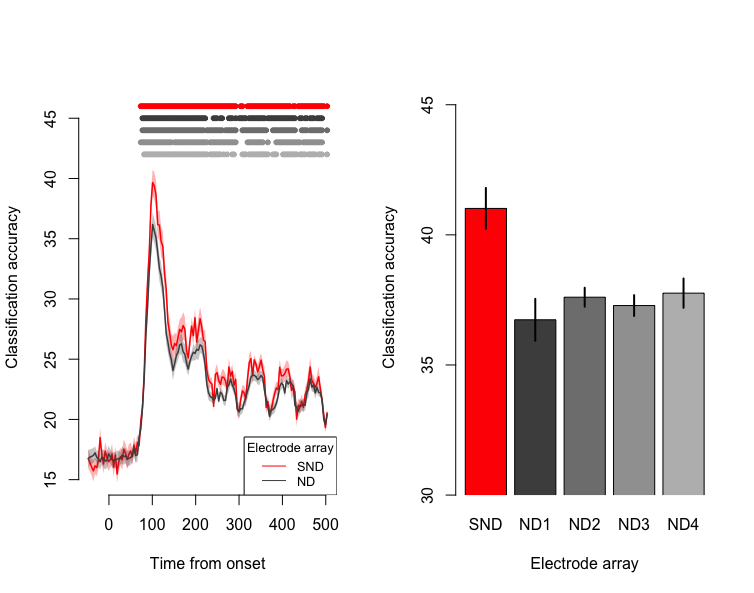


Figure S5.2. Decoding results across time. SND decoding was significantly greater than the interpolated ND arrays. (a) Mean accuracy for 6-way classification, performed for each time point for the SND and mean of interpolated ND electrode arrays. Shaded area depicts standard error of the mean corrected for within subjects designs. Dots depict time points with significant group-wise decoding per array, *p* < .001. (b) Mean decoding accuracy per electrode array, 10 ms around each individual’s first peak in decoding. SND outperformed all interpolated ND arrays. Interpolation did not appear to influence decoding (c.f. Figure 2 of paper).

*Frequency domain decoding*

Decoding between the six experimental stimuli using the SSVEP amplitude at 15 Hz with the interpolated ND arrays yielded the same trend of results as the original, smaller ND sets; SND outperformed ND (see Figure S5.2a). A two way ANOVA with factors of electrode array and percent PCA features revealed a significant effect of electrode array, *F*_4,60_ = 9.99, *p* < .001,  *η*_G_^2^ = .262, with SND outperforming each ND array, *t*s > 3.00, *p*s < .003, *g*_av_ > .483. There was also a significant main effect of the number of features fed to the classifier (i.e., PCA components), *F*_1,15_ = 34.31, *p* < .001,  *η*_G_^2^ = .446. The interaction between electrode array and features did not reach significance, *F*_4,60_ = 1.78, *p* = .144,  *η*_G_^2^ = .014. Therefore, once again the SND array outperformed the ND arrays, even when the number of electrodes was equivalent.

Figure S5.2b shows the percent variance accounted by the PCA components. As expected, as the number of PCA components increased, so did the percent variance accounted for by those components. Note that the interpolated ND datasets contained more meaningful PCA components than the number of original electrodes (~60 components accounting for 99% of the variance coming from 30-33 ND electrodes). Importantly, however, the number of ND components accounting for 99% of the variance did not reach as high as the SND dataset, supporting the claim that the SND array contained more meaningful neural information.


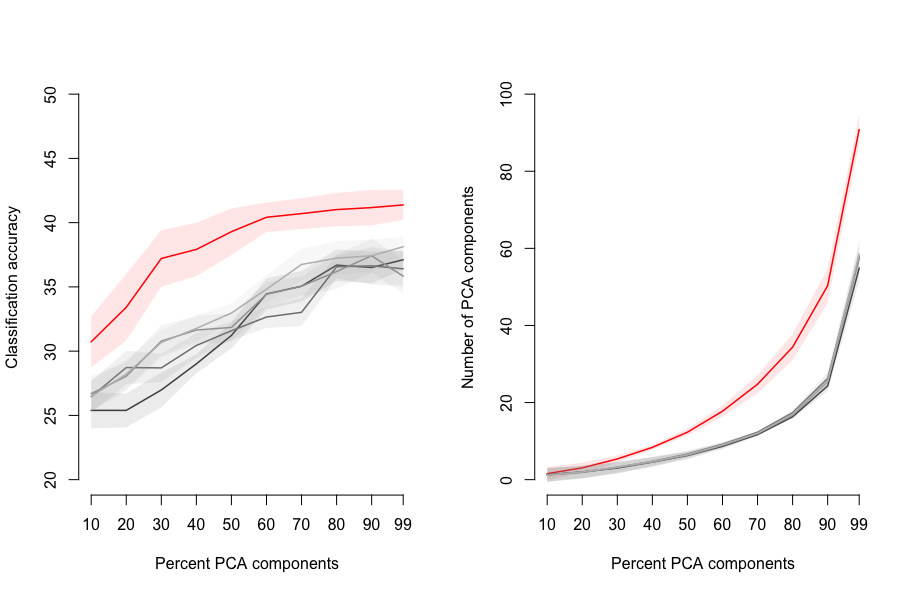


Figure S5.3. Decoding accuracy for frequency domain data with interpolated ND datasets. (a) Classification accuracy as function of the number of PCA components fed to the classifier per electrode array. SND had the best decoding accuracy for every level of PCA components. (b) Number of PCA components as a function of the percent variance accounted for by the PCA components. These were the number of features used for classification.

*Representational similarity analysis (RSA)*

The RSA analysis across time revealed that the SND array had a significantly higher correlation with the HMAX S1 model than the interpolated ND arrays (Figure S5.4). At the time of maximum correlation per participant, there was a significant effect of electrode array, *F*_4,60_ = 3.02, *p* = .024, *η*_g_*^2^* = .077. SND was significantly higher than all ND arrays, *t*s > 2.38, *p*s < .031, *g*_av_ > .575 (Figure S5.4b). There were no significant differences across ND arrays, *t*s < 1.26, *p*s > .227, *g*_av_ < .219.

As can be see in Figure S5.4c, the Fourier plot of the correlation results revealed peaks at 15 Hz and its harmonics for the SND and interpolated ND sets, as in the original analysis. There was a marginal effect of array on the 15 Hz signal, *F*_4,60_ = 2.17, *p* = .083, *η*_g_*^2^* = .031, such that the amplitude was significantly higher for the SND than the ND1, ND3 and ND4 arrays, *p*s < .030. SND was higher than ND2, but this did not reach significance, *p*s = .248. There were no significant differences across ND arrays, *p*s > .226. This analysis therefore confirmed that SND outperformed the ND arrays even when the ND sets were equalized for number of electrodes. The correlations, in fact, were numerically lower for the interpolated datasets than the original smaller ND arrays, indicating that the interpolated channels might have contributed some noise to this analysis.

Taken together, the analysis of the interpolated ND arrays showed that SND consistently outperformed the ND electrode arrays for classification and representational similarity analysis. The SND advantage is therefore not reliant on the number of electrodes per se, but the additional neural information obtained by sampling at higher electrode density.


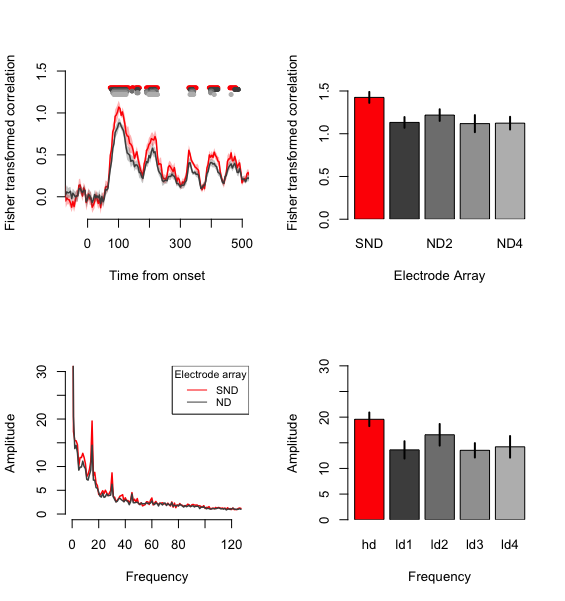


Figure S5.4 RSA analyses with SND and interpolated ND datasets. SND had significantly higher correlation with the HMAX S1 model than the interpolated ND arrays. (a) Correlation between HMAX S1 and EEG stimulus dissimilarity across time for each electrode array (high density and mean interpolated low density). Dots signify significant groupwise correlations per array. (b) Mean correlation for each electrode array, 10 ms around each individual’s first peak within the time period 80-140 ms. SND was significantly more correlated than interpolated ND arrays. (c) Fourier transform of RSA correlation values 0-1000 ms. (d) Mean 15 Hz signal in correlation values per electrode array.

Supplementary references

Bigdely-shamlo, N., Mullen, T., Kothe, C., Su, K., & Robbins, K. A. (2015). The PREP pipeline : standardized preprocessing for large-scale EEG analysis. *Frontiers in Neuroinformatics*, *9*. http://doi.org/10.3389/fninf.2015.00016

Sandwell, D. T. (1987). Biharmonic spline interpolation of GEOS-3 and SEASAT altimeter data. *Geophysical Research Letters*, *14*(2), 139–142.

Woodhead, Z. V. J., Wise, R. J. S., Sereno, M., & Leech, R. (2011). Dissociation of sensitivity to spatial frequency in word and face preferential areas of the fusiform gyrus. *Cerebral Cortex*, *21*(10), 2307–2312. http://doi.org/10.1093/cercor/bhr008
